# Immersive hand hygiene trainer for physicians – a story-based serious game

**DOI:** 10.1186/1753-6561-5-S6-O31

**Published:** 2011-06-29

**Authors:** HS Sax, Y Longtin

**Affiliations:** 1 Infection control program, University of Geneva Hospitals and Medical Faculty, Geneva, Switzerland; 2 Infectious diseases, Centre Hospitalier Universitaire de Québec, Québec, Canada

## Introduction / objectives

Hand hygiene is a simple yet often omitted gesture to prevent pathogen transmission and healthcare-associated infections. Training healthcare workers in hand hygiene is a key element in any multifaceted promotion strategy. Physicians are notoriously known for their underperformance in this field. We sought to design a natural immersive environment to improve physicians’ hand hygiene performance.

## Methods

We inserted filmed sequences based on a plot of two physicians interacting with different patients during ward rounds into an interactive computer interface allowing the physician 'gamer' to decide where to use hand hygiene and disposable gloves. Hand hygiene being a very repetitive and often subconsciously executed task, virtual immersion might increase learning and improve long-term retention. Thus, we used both an emotionally engaging but also distracting plot to create role identity and simulate mental load typical for medical activity on the ward. Design features were refined through individual think-aloud protocols and target group testing. Immediate feedback messages and a result tracking mechanism were added.

## Results

The design specifications could all be met. The resulting application proofed equally suitable for the training of hand hygiene observers. Computing the user’s results allows for benchmarking.

## Conclusion

A serious game was successfully launched immersing the ‘gamer’ into the real-life challenge of hand hygiene. Post-launch evaluation and clinical effectiveness have to be performed in a next step.

## Disclosure of interest

None declared.

